# Esophageal bypass surgery as a definitive repair of recurrent acquired benign bronchoesophageal fistula

**DOI:** 10.1186/s13019-019-0902-2

**Published:** 2019-04-11

**Authors:** Jiri Vachtenheim, Robert Lischke

**Affiliations:** Third Department of Surgery, First Faculty of Medicine, Charles University Prague and University Hospital Motol, Prague, Czech Republic

**Keywords:** Acquired benign bronchoesophageal fistula, Esophageal stent, Esophageal bypass surgery

## Abstract

**Background:**

Acquired benign bronchoesophageal fistula (BEF) is rare and develops as a complication of other diseases, mostly of inflammatory processes and traumas of the chest. The treatment of choice is a surgical repair, which is considered definitive and leads to successful outcomes. However, incidence of recurrence after the primary repair based on limited data is up to 10% and its treatment is challenging. We report a surgical case of a patient with recurrent acquired benign BEF after primary resection and ensuing successful definitive repair with esophageal bypass surgery after temporary esophageal stenting.

**Case report:**

A 46-year-old male was referred to our department with a symptomatic left-sided bronchoesophageal fistula as a complication of severe acute necrotizing mediastinitis that originated from odontogenic abscess. Previously, several cervicotomies and bilateral thoracotomy were performed at an external medical facility to manage the acute condition. We performed resection of the fistula through re-thoracotomy. Postprocedural esophagography demonstrated a recurrence of bronchoesophageal communication. Postinflammatory adhesions excluded further repair through thoracotomy, therefore a stent was introduced in the esophagus for 12 weeks. Thereafter, an esophageal bypass surgery using a substernaly interposed gastric conduit was performed and resulted in an excellent long-term outcome.

**Conclusions:**

Esophageal bypass surgery using a substernaly interposed gastric conduit may be considered if the standard surgical repair of acquired benign bronchoesophageal fistula is not successful or feasible.

## Background

Acquired benign bronchoesophageal fistula (BEF) is a rare pathology, which develops as a complication of other diseases, mostly of inflammatory processes and traumas of the chest. Due to the non-specific symptoms of BEF, the diagnosis is often delayed and hence the treatment is inadequate. Despite progress in endoscopic methods, non-operative treatment of benign BEF is generally unsatisfactory. The treatment of choice is a surgical repair, which is considered definitive and leads to successful outcomes [1, 2]. However, surgery is associated with the adverse events, of which the most feared one is a recurrence of the fistula. Any subsequent open repair with thoracotomy is technically challenging and implies a high risk of complications [3]. The incidence of recurrence after the primary surgery based on limited data is up to 10% [1–4]. The type of surgical treatment of recurrent acquired benign BEF is an intensely debated subject. Here we report an ineffective primary surgical repair of acquired benign BEF as a rare complication of necrotizing mediastinitis that originated from odontogenic abscess and ensuing successful definitive repair with esophageal bypass surgery after temporary esophageal stenting.

## Case presentation

**Fig. 1:**
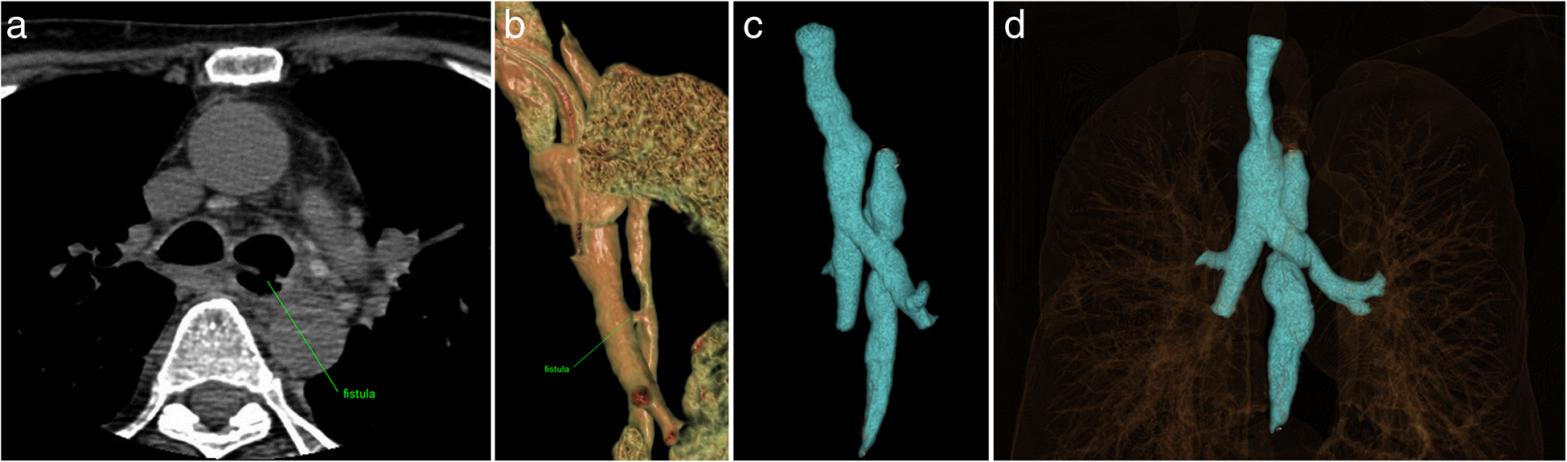
Bronchoesophageal fistula before primary resection and after esophageal bypass surgery. **a** Preoperative computed tomography revealing fistula between esophagus and left main bronchus, approximately 1 cm from tracheal bifurcation (green arrow). **b** Preoperative three-dimensional rendering of fistulous tract (green arrow). **c, d** Three-dimensional rendering 5 months after the esophageal bypass surgery revealing esophagus resected above and below the bronchoesophageal fistula level, leaving this section in situ on the posterior wall of the left main bronchus as a neo-pseudo-diverticulum. Substernaly interposed gastric conduit is not shown

A 46-year old patient with a history of complicated descendent purulent mediastinitis that originated from odontogenic abscess underwent several surgical interventions (cervicotomies and bilateral thoracotomy) at another medical facility. The patient’s condition was complicated by the development of multiple organ dysfunction syndrome, with the necessity to introduce tracheostomy, percutaneus endoscopic gastrostomy (PEG) and amputation of distal phalanges of the right foot due to septic embolization. After the management of the acute condition, further course was complicated by development of fistula between esophagus and left main bronchus approximately 1 cm from tracheal bifurcation presenting with recurring polyresistant bronchopneumonias (Fig. 1a-b). The patient was incapable of oral intake because of aspirations and cough. The patient was transferred to our department with request for surgical repair. During examination, he presented with non-productive cough, mild anaemia and depleted physiological reserves. The patient was in a poor nutritional condition (body mass index 16,7), but his general condition was sufficient for surgery and we decided to perform a primary surgical repair.

Due to the necessity of selective ventilation of the right lung only, which was also affected by the chronic inflammatory process, the procedure was conducted with peripheral veno-venous extracorporeal membrane oxygenation (VV-ECMO) support. The fistula was resected through the right re-thoracotomy and both bronchial and esophageal defects were sutured in one (with soft tissue underlay), and two layers, respectively.

**Fig. 2:**
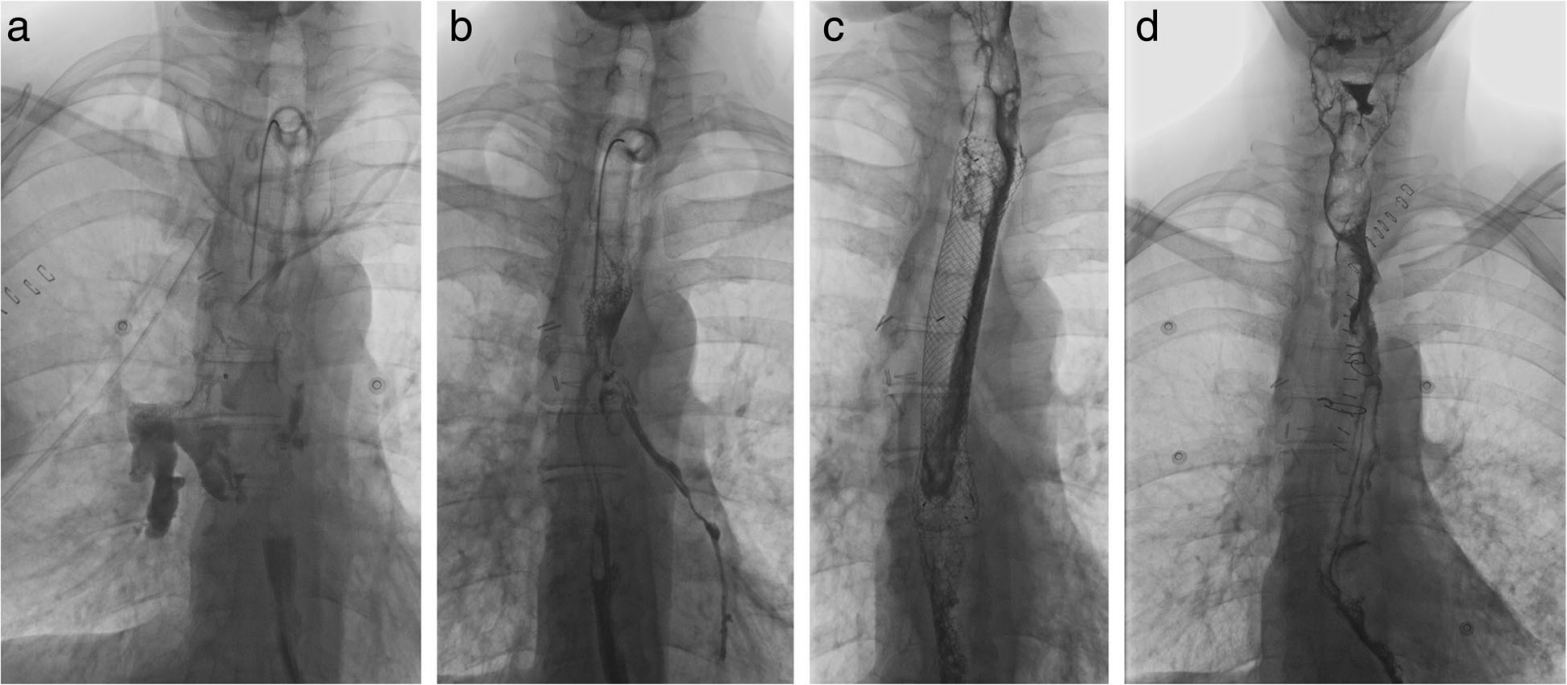
Esophagography findings. **a** Leak in esophageal suture 1 week after primary repair. **b** Recurrence of bronchoesophageal fistula on postoperative day 18 after primary repair. **c** Temporarily introduced self-expandable metallic stent sufficiently sealing the recurrent bronchoesophageal fistula 8 weeks after primary surgery. **d** One week after esophageal bypass surgery with no signs of extralumination or stagnation of contrast agent

One week after the surgery, esophagography demonstrated a leak in the esophageal suture (Fig. 2a). Due to the low inflammatory parameters and effective drainage of the right hemithorax through a chest drain, conservative procedure was chosen. After next 11 days esophagography revealed regular recurrence of bronchoesophageal communication (Fig. 2b). As postinflammatory adhesions excluded further repair through thoracotomy, a self-expandable metallic stent (SEMS) was introduced into the esophagus as a temporary bridge to definitive surgical solution.

**Fig. 3:**
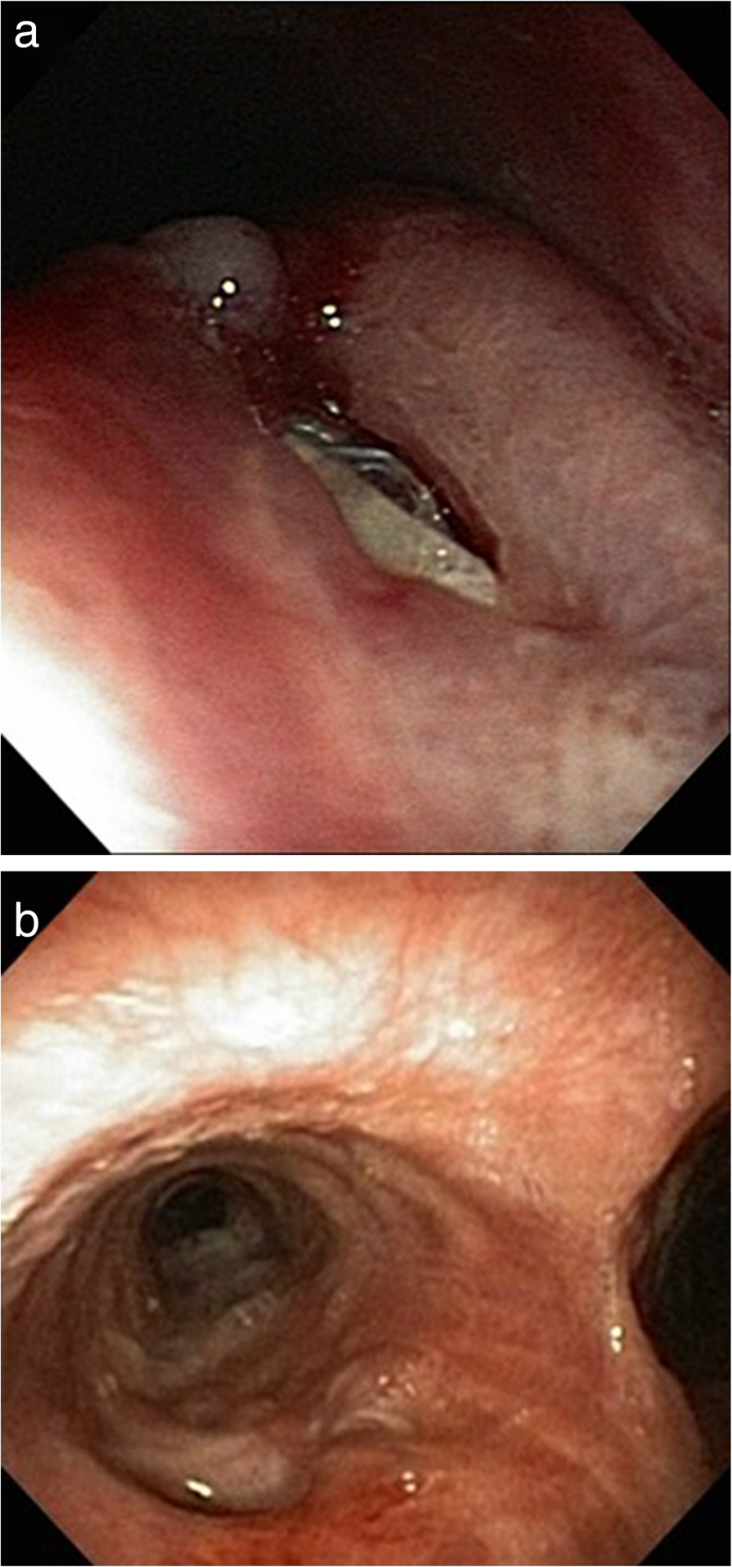
Bronchoscopic findings. **a** Bronchoesophageal fistula in left main bronchus with gaping esophageal stent 13 weeks after primary surgery. **b** Blind asymptomatic fistula with non-inflammatory mucous membrane 5 months after esophageal bypass surgery

With the SEMS, the patient well tolerated orally administered fluids. He was discharged 9 days after stent introduction in a stable condition with a defined nutritional regimen. The follow-up esophagography showed a satisfactory position of SEMS sufficiently sealing the recurrent BEF (Fig. 2c). The persisting BEF with a gaping SEMS in the left main bronchus was verified bronchoscopically (Fig. 3a). Prior to definitive surgical repair, patient’s overall condition was carefully evaluated. The nutritional status was in mild anabolic phase. Echocardiography, microbiological cultures and hemocultures did not prove any contraindication for surgery. Four months after the unsuccessful primary surgical procedure, esophageal bypass surgery through upper abdominal incision and left lateral cervicotomy with resection of left sternoclavicular joint was conducted. We performed a resection of the esophagus above and below the bronchoesophageal fistula level, leaving this section of the esophagus in situ on the posterior wall of the left main bronchus as a neo-pseudo-diverticulum. Then, cervical end-to-side gastroesophageal anastomosis in two layers using substernaly interposed gastric conduit was constructed. During the surgery, PEG was removed and feeding jejunostomy was carried out, which was later also removed.

Esophagography has not revealed extralumination or stagnation of the contrast agent 1 week after surgery (Fig. 2d). Subsequent realimentation was well tolerated by the patient. Further postoperative course was uneventful. The patient was discharged 2 weeks after the esophageal bypass surgery in very good condition. Follow-up examinations demonstrated favourable findings (Fig. 1c-d, Fig. 3b). The patient had no symptoms after eating or any other problem for the 2-year follow-up period. The esophageal remnant did not result in any inflammatory or other complications. Patient returned to his work after recovery and almost achieved his normal weight.

## Discussion

Acquired benign BEF is a rare pathology representing complex clinical problem. Despite the biologically benign nature of the condition, the course of the disease may be fatal. Successful treatment requires a multidisciplinary approach and specific course of action differs case by case. Although several reports of primary endoscopic treatment of BEF were published, first-line treatment remains surgical repair which results in excellent long-term outcomes and should be performed as soon as possible after diagnosis [3, 4]. The timing of the surgery is also given by the patient’s condition. Among the basic initial actions are the elimination of oral intake, elevation of the bed under the head by at least 45 degrees, anti-reflux treatment and aspiration from the oral cavity. Prior to the procedure, it is often necessary to improve the nutritional parameters with high quality parenteral nutrition and control of sepsis. The same applies to cases where a specific etiologic agent is known and it should be eradicated before the operation [5]. The preoperative preparation and the operation itself should include the management of aspiration, regular bronchoscopic routine and the management of gastric reflux using a nasogastric tube [6].

For patients in critical condition or patients that are unable to undergo the surgery immediately, it is appropriate to introduce a SEMS into the esophagus to seal the fistula as a bridge to surgical repair. SEMS is otherwise considered to be treatment of choice in malignant fistulas [7]. In our case, patient’s general condition was sufficient for surgery, therefore temporary esophageal stent introduction was not considered.

From the perspective of anaesthesiology, a major problem during the surgery is the management of airways and adequate oxygenation and carbon dioxide elimination. Selective ventilation of the non-affected lung is necessary. A legitimate option is to avail ECMO support [8].

The standard and generally the most recognized surgical treatment of an acquired benign BEF is thoracotomy (mostly right-sided) with identification, exposure and resection of the fistula. Then suture of the esophagus in two layers and suture of the bronchus with interposition of pedicled tissue between the sutured esophagus and bronchus is performed [1]. It is generally accepted that the use of soft tissue (posteriorly pedicled intercostal muscle flap, omentum, pericardial flap, pleural flap, diaphragmatic muscle flap) as the support tissue to separate the esophageal and bronchial sutures reduces the risk of recurrence [1, 5, 9]. In our case, we preferred this procedure to the directly performing esophageal bypass surgery with respect to the condition of the patient and the intention of shorter and less burdensome surgery. However, this procedure didn’t result in satisfactory outcome as an early postoperative recurrence of BEF has occured, probably due to severe postinflammatory terrain. Therefore, temporary esophageal stent was introduced. After 12 weeks, successful esophageal bypass surgery was performed, resulting in an excellent long-term outcome.

Recurrence of BEF after primary surgical repair is not frequent but challenging and the type of surgical treatment is an intensely debated subject. Ultimately, the condition can be solved with cervical esophagostomy and nutritive gastrostomy. This approach, however, negatively affects the quality of life. To the best of our knowledge, the case presented herein is the first report describing a recurrent acquired benign BEF in an adult that was resolved by esophageal bypass surgery. This procedure is otherwise reserved and well recognized palliative surgical treatment for fistulas of malignant aetiology [10].

## Conclusions

Esophageal bypass surgery may be considered if the standard surgical repair of acquired benign BEF is not successful or feasible. In addition to surgical treatment, the case demonstrates an essential significance of the interdisciplinary approach and use of other supportive methods, such as perioperative ECMO or temporary esophageal stenting in the complex treatment of BEF.

## Abbreviations

BEF: Bronchoesophageal fistula
PEG: Percutaneus endoscopic gastrostomy
SEMS: Self-expandable metallic stent
VV-ECMO: Veno-venous extracorporeal membrane oxygenation
